# The Hitchhiker's guide to isolated organ perfusion: a journey to 2040

**DOI:** 10.3389/frtra.2025.1642724

**Published:** 2025-08-13

**Authors:** John Fallon, Alex Sagar, Mohamed Elzawahry, Hatem Sadik, Kazuyuki Gyoten, Syed Hussain Abbas, Richard Dumbill, Peter Friend

**Affiliations:** The Nuffield Department of Surgical Sciences, University of Oxford, Oxford, United Kingdom

**Keywords:** Organ Perfusion, Preservation, NMP, HMP, HOPE

## Abstract

Building on the established success of hypothermic machine perfusion (HMP) and emerging normothermic platforms, machine perfusion is poised to guide a journey toward 2040, transforming organ transplantation into an era of integrated preservation, viability assessment, and ex situ therapy. While renal HMP today reduces delayed graft function and improves graft survival, the next two decades will centre on adaptive platform trials in normothermic perfusion, predictive AI-driven biomarkers, and unified registries to validate robust surrogate endpoints. Centralised Assessment and Reconditioning Centres (ARCs) will streamline 24/7 workflows, combining advanced imaging, molecular assays, and gene or cell therapies to repair and optimise grafts ex-vivo. Health economics will shift toward dynamic, value-based reimbursement, addressing equity and cost-effectiveness across diverse systems. Regulatory frameworks will adapt through CONSORT-style reporting and direct device-to-registry data integration, ensuring transparency and reproducibility. By 2040, these convergent advances in HMP, normothermic machine perfusion (NMP), along with translational research will not only enhance graft utilisation and patient outcomes but will redefine transplantation paradigms through precision graft management, optimised logistics, and new indications such as extracorporeal organ support.

## The state of the art

The current landscape of organ perfusion is a spectrum from hypothermia to normothermia, and from routine clinical use underpinned by multiple randomised controlled trials (RCTs) to exploratory preclinical animal studies, with considerable international variation in both uptake and specific protocols. Perfusion in essence is divided by the temperature of the perfusate and whether that perfusate is oxygenated. Although various nomenclatures have been used to describe different modalities of perfusion, current practice is converging on the use of the following: hypothermic machine perfusion (HMP) describes cold perfusion without oxygenation; hypothermic oxygenated machine perfusion (HOPE) describes circuits in which oxygen is delivered; controlled oxygenated rewarming (COR) describes a process in which an organ is transitioned progressively from hypothermia to normothermia; normothermic machine perfusion (NMP) describes perfusion with warmed, oxygenated, usually blood based perfusate at normal body temperature. In contrast to these ex-situ isolated organ perfusion methodologies, normothermic regional perfusion (NRP) delivers *in situ* perfusion, in which the abdominal (or thoraco-abdominal) organs are perfused immediately following circulatory determination of death prior to surgical retrieval. NRP is outside of the scope of this review, but is increasingly used in combination with either HOPE or NMP.

Isolated organ perfusion technology has been applied extensively in both abdominal and thoracic organs (Table 1). Renal HMP is the most established implantation: in many countries this is standard of care, and supported by evidence from studies and meta-analyses of over 16 RCTs, demonstrating reduced delayed graft function (DGF), and improved graft survival (2, 35–40). Within this area there are several devices and perfusates available. The LifePort™ Kidney Transporter (Organ recovery Systems, Inc. Itasca, IL, USA) and the XVIVO Kidney Transporter™ (XVIVO Perfusion AB. Gothenberg, Sweden) are the current market-leading portable devices. Commercially available devices are portable and easy to use, allowing uncomplicated use from the point of retrieval (“device-to-donor”) without the need for highly specialised expertise (i.e., a dedicated perfusionist). Notably, there has been a trend in recent years towards to the delivery of oxygen, with good evidence that the delivery of oxygen is beneficial, even at cold temperatures.

**Table 1:** The randomised perfusions studies and notable non-randomised studies for each of the organs, divided by perfusion termerature and oxygenation.

Organ	First author	Year	Randomised	Modality	Perfusion timing	Device	O_2_	Patient no.	Primary endpoint(s)	Key outcome(s)	Funding/health–system
Kidney	van der Vliet (1)	2001	No	HMP vs. SCS	Not specified	Gambro PP	No	76	DGF	Decreased DGF	Public (Dutch Zorgverzekeraars)
Kidney	Moers (2)	2009	Yes	HMP vs. SCS	Continuous	LifePort	No	752	DGF	Decreased DGF; Improved graft survival	Mixed (Dutch MoH & Organ Recovery Systems)
Kidney	Watson (3)	2010	Yes	HMP vs. SCS	Mix	LifePort	No	90	DGF	No difference	Public (UK NHSBT/NIHR)
Kidney	Wszola (4)	2013	Yes	HMP vs. SCS	Continuous	LifePort vs. RM3	No	50	1y eGFR	Noninferior	Public (Polish state grant)
Kidney	Tedesco-Silva (5)	2017	No	HMP vs. SCS	Continuous	RM3	No	160	DGF	Decreased DGF	Not reported—Brazil
Kidney	Wang (6)	2017	Yes	HMP vs. SCS	Continuous	LifePort	No	48	DGF	No difference	Public (China National Natural Science Foundation)
Kidney	Zhong (7)	2017	Yes	HMP vs. SCS	Continuous	LifePort	No	282	3 y graft survival	Improved survival	Public (China National Foundation)
Kidney	Summers (8)	2020	No	HMP vs. SCS	Continuous	LifePort	No	102	DGF	No difference	Public (UK NHSBT)
Kidney	Husen (9)	2021	Yes	HOPE 4h vs. SCS	End ischaemic	Kidney Assist	Yes	262	1 y graft survival	Neutral; trend improved	Public (Eurostars/FP7 Euro-HOPE)
Kidney	Jochmans (10)	2020	Yes	HOPE vs. HMP	Continuous	Kidney Assist	Yes	212	1y eGFR	Improved eGFR; Decreased rejection	Public (EU Horizon 2020 grant 732035)
Kidney	Zlatev (11)	2022	No	COR	End ischaemic	Kidney Assist	Gradual	6	Day 7 CrCl	Improved CrCl	Public (German DFG)
Kidney	Hosgood (12)	2023	Yes	NMP 1 h	End ischaemic	Custom circuit	Yes	277	DGF	No difference	Public (UK NIHR/NHSBT)
Kidney	Dumbill (13)	2025	No	NMP 2–24 h	End ischaemic	OrganOx Kidney	Yes	36	30d graft survival	100% survival; no PNF	Public (UK NIHR)

Liver	Dutkowski (14)	2015	No	HOPE vs. SCS	End ischaemic	Liver Assist	Yes	50	EAD	Decreased EAD	Public (Swiss SNF)
Liver	Van Rijn (15)	2018	No	HOPE vs. SCS	End ischaemic	Liver Assist	Yes	30	Biliary injury score	Lower injury score	Public (Dutch Transplant Foundation)
Liver	Van Rijn (16)	2021	Yes	HOPE vs. SCS	End ischaemic	Liver Assist	Yes	160	Biliary stricture	Lower stricture rate	Public (Fonds NutsOhra Charity)
Liver	Czigany (17)	2021	Yes	HOPE vs. SCS	End ischaemic	Liver Assist	Yes	109	Peak AST	Lower peak AST	Not reported - Europe
Liver	Ravaioli (18)	2022	Yes	HOPE vs. SCS	End ischaemic	Vitasmart	Yes	110	EAD	Decreased EAD	Public (Italian Ministry of Health)
Liver	Panayotova (19)	2024	Yes	HOPE vs. SCS	Continuous	LifePort Liver	Yes	179	EAD	Non-inferior	Industry (Organ Recovery Systems)
Liver	Schlegel (20)	2023	Yes	HOPE vs. SCS	End ischaemic	Liver Assist	Yes	203	Major complications	Decreased complications	Public (Swiss National Science Foundation)
Liver	Minor (21)	2022	Yes	COR	End ischaemic	Custom circuit	Gradual	20	Peak AST	Lower peak AST	Public (German DFG)
Liver	Ravikumar (22)	2016	Yes	NMP	Continuous	OrganOx	Yes	20	EAD	No difference	Public (UK NIHR Efficacy award)
Liver	Nasralla (23)	2018	Yes	NMP	Continuous	OrganOx Metra	Yes	220	EAD	Decreased EAD; Increased utilisation	Public (UK MRC & NIHR)
Liver	Ghinolfi (24)	2019	No	NMP	Continuous	OCS Liver	Yes	10	Utilisation	All allografts met viability criteria	Mixed (Pisa Hospital & TransMedics Inc)
Liver	Quintini (25)	2021	No	NMP	Continuous	Custom circuit	Yes	15	EAD	Decreased EAD	Public (Cleveland Clinic)
Liver	Markman (26)	2022	Yes	NMP	Continuous	OCS Liver	Yes	293	EAD	Decreased EAD	Industry (TransMedics Inc)
Liver	Chapman (27)	2023	Yes	NMP	Continuous	OCS Liver	Yes	266	EAD	Neutral overall; benefit high-risk	Industry (TransMedics Inc)
Liver	Guo (28)	2023	Yes	NMP	Continuous (ischaemia-free)	Custom circuit	Yes	65	Utilisation	Increased utilisation	Public (China National NSF)
Liver	Mergental (29)	2020	No	NMP	End ischaemic	OrganOx	Yes	22	Successful Tx	100% transplanted	Public (UK Birmingham University/QEHB Charity)

Heart	Ardehali (30)	2015	Yes	NMP	Continuous	OCS Heart	Yes	130	30d graft failure	Noninferior to SCS	Industry (TransMedics Inc)

Lung	Fisher (31)	2016	No	EVLP (NMP)	End ischaemic	XVIVO XPS (Toronto protocol)	Yes	24	1-y graft and patient survival	EVLP non-inferior but cost–effectiveness marginal	Public (UK NIHR Health Technology Assessment Programme)
Lung	Slama (32)	2017	Yes	EVLP (NMP)	End ischaemic	XVIVO XPS (Toronto protocol)	Yes	80	PGD grade 3 at 72 h	No harm; trend to less PGD, similar 1y survival	Public (Austrian Research Fund/Vienna Lung Trials)
Lung	Warnecke (33)	2018	Yes	EVLP (NMP)	Continuous	OCS Lung	Yes	317	PGD3 within 72 h	Noninferior; Increased utilisation	Industry (TransMedics Inc)
Lung	Loor (34)	2019	No	EVLP (NMP)	Continuous	OCS Lung	Yes	79	30-day patient & graft survival (safety)	No safety concerns	Industry (TransMedics Inc)

Colour coded according to HMP, HOPE, COR or NMP, with device, perfusion timing key outcome and funding source.

This contrasts with heart perfusion in which there are two small non-randomised studies utilising HOPE for a total 16 cardiac recipients (41, 42), demonstrating safety and feasibility. Lung perfusion has been a focus of much research over many years with a clear current consensus in favour of normothermic oxygenated perfusion (EVLP) rather than HMP or HOPE. Normothermic EVLP has been utilised in large, randomised trials, with increased lung utilisation and noninferior outcomes. HMP had very limited interest until very recently, confined to a rat study in the published literature (43). However recently a single phase 1 clinical study has been reported suggesting that HOPE used in combination with EVLP is safe and feasible (44).

For intestinal and pancreas preservation, HMP remains confined to large animal models currently (45, 46). Encouraging pre-clinical work in pancreas perfusion for preservation (45–50) and islet isolation (51, 52) has resulted in a pancreas a first in-man HMP trial scheduled to start soon (53).

Although not as extensive as in the kidney, there is an increasing body of evidence and clinical experience of hypothermic liver perfusion, almost entirely utilising HOPE, although with differing methods of delivering oygen to the perfusate: Liver Assist™ (XVIVO Perfusion AB), LifePort™ Liver Transporter (Organ Recovery Systems Inc.) and VitaSmart™ Machine Perfusion System (Bridge to Life Ltd) a the current commercial devices. Multiple RCTs (*n* = 6) show benefits with respect to early allograft dysfunction, post reperfusion syndrome, post operative complications, ICU/hospital stay and later biliary complications (16, 17, 19, 20, 54, 55). Two metanalyses show improvement of graft and patient survival (56, 57). The benefits of perfusion are particularly notable in the recipients of higher-risk donor organs, including those organs from donors declared dead by cardiovascular criteria (DCD).

Across all organs there is increasing consensus in favour of the delivery of oxygen, but there is less consensus regarding the issue of temperature. Normothermic systems are characterised by higher complexity (and therefore cost), and there is much current debate as to the extent to which these disadvantages are outweighed by other benefits. The primary arguments in favour of NMP are that this not only lengthens safe preservation times (reducing logistic constraints) but also allows more comprehensive pre-transplant assessment of the organ (which assists in deciding whether an organ is transplantable) and thereby optimises the utilisation of higher-risk organs.

Hypothermia and normothermia present two fundamentally different approaches to the challenge of organ preservation. The intention of hypothermia is to reduce the rate of cellular metabolism and therefore the need for energy substrates, whilst preventing the ill-effects of metabolic accumulation. Cellular metabolism does not cease at ice temperature, but it is reduced to around 10% of the physiological rate. Mitochondrial energy production in anoxic conditions leads to the accumulation of succinate, and this is rapidly metabolised at the time of reperfusion, leading to the production of reactive oxygen species that are a major cause of ischemia-reperfusion injury (IRI). In a fundamental discovery, which now underpins current thinking in hypothermic organ preservation, Chouchani et al. (58) showed that this process can be abrogated even under hypothermic conditions by providing oxygen. It is this important process that is exploited in HOPE, and believed to be the primary reason for the reduction in the manifestations of IRI in HOPE settings in all organ types.

In contrast to HMP/HOPE, NMP is designed to replicate (as near as possible) normal physiology. The unifying aim to support metabolic function has led to the broadening interest in normothermic perfusion. Maintenance of a near physiological milieu theoretically removes the correlation between preservation duration and outcome. This allows longer preservation duration and relieves logistic constraints. At its most basic, NMP allows a functioning organ to be tested in numerous ways and to reverse the effects of hypoxia. This is the reason for its recent success within liver transplantation. There are currently two FDA approved devices, (manufactured by OrganOx & OCS™), which have shown to benefit logistics, utilisation and outcomes (23, 24, 26–28) .In particular, NMP has shown improved utilisation of organs discarded on the basis of conventional criteria, demonstrating long term patient survivals which are comparable to outcomes with standard grafts (29, 59). Its prevalence has reached a point that now in the US nearly 1 in 5 livers undergoes NMP (60).

Improving logistics and utilisation similarly underly the benefit of NMP above the diaphragm. The two FDA-approved lung perfusion devices (OCS™ & XVIVO™) for EVLP have generated strong RCT data (32, 33), and non-randomised US & UK studies have demonstrated successful utilisation of higher risk donors or those declined by standard criteria organs, leading to significantly shorter waiting times and fewer deaths on the waiting list (31, 34, 61, 62). In heart transplantation, the OCS device used in the multi-centre US PROCEED II trial demonstrated feasibility and safety, with significantly longer overall preservation times, up to 9 h (30).

Extension of preservation times in liver transplantation is increasingly seen as an important benefit, and some liver units that have adopted this technology no longer (or rarely) perform overnight implantation (63, 64). As with the introduction of many new technologies, early adopters are exploring its use outside the limits approved by regulators [e.g., in the most extreme case reported to date, a liver was successfully transplanted after being preserving for 72 h (65)]. While perfusion durations of this extreme length for logistical reasons are likely to remain rare, there are novel indications which may warrant such lengths—for example biliary epithelial regeneration in injured grafts, which has been seen in one pre-clinical study involving prolonged perfusion for up to 13 days (66).

Renal NMP trials have also been conducted, but a clear rationale for renal NMP has yet to be established particularly given that lack of immediate graft function is inherently less problematic than in liver, lung or cardiac transplantation. Although clinical studies in renal NMP have been published describing short and prolonged duration of perfusion, these have not yet demonstrated benefit in terms of reduction in DGF or improvement in longer-term function (12, 13, 67). As with liver and cardiothoracic transplantation, the real driver of this technology is likely to be the improvement in the utilisation of higher-risk organs. In the UK alone, “poor perfusion” accounts for nearly 20% of the roughly 350 annual organ discards after retrieval. For the US, even accounting for population size, the figures are more striking—over 8,000 kidneys were discarded post-retrieval in 2023 (68). There could be substantial utilisation benefit if these organs were subjected to a more thorough evaluation based on HMP or NMP, and if the adverse effect of prolonged storage was minimised. Only once large numbers of transplants have been performed following NMP, however, will it be possible to elucidate rigorous organ assessment criteria for the kidney.

As perfusion technologies become more established, diversification will occur exploring different ways to exploit the technology. Within liver transplantation, graft modification [e.g., defatting livers (69), gene therapy etc.], liver splitting and extra-corporeal liver cross-circulation are on the horizon.

Xenotransplantation may be imminent and will likely accentuate the need for better methods of organ preservation. The location and number of clinical porcine production facilities will be highly limited. Porcine organs tolerate cold ischaemia poorly. Although these organs are, in essence, from living donors, these are also juvenile as well as a different species. Much remains to be learned regarding the longer-term behaviour of xeno-organs, and it is likely that the avoidance of transplant-related damage (especially IRI) will be of great importance. For example, the propensity to vascular injury and fibrosis may increase the need for optimum organ preservation methods.

## Trial design, reporting, registries, & regulation

Isolated organ perfusion is a complex, multi-parametric intervention covering a heterogenous array of techniques. The breadth of different approaches is exemplified by the early clinical literature on isolated kidney perfusion—no groups used the same devices/ perfusate composition or agree on how best to implement the logistics of perfusion. Key differences include perfusate composition (e.g., whether there is a protein compartment to provide colloid oncotic pressure); whether arterial pressure is applied continuously or with a pulsatile waveform (70). This protocol heterogeneity underlies a need for a modern approach to trial design, which is flexible to perfusion parameter modulation within the scope of a single trial and permits rapid iteration. Platform trial designs are well-suited to this problem and are gaining popularity in other fields (71). However, a particular problem that isolated organ perfusion faces is that transplantation remains a relatively low-volume activity, meaning that the number of patients available to recruit to such trials is limited. Also, with the benefit of modern surgical techniques and immunosuppression, transplantation is also extremely successful as a treatment modality, with respect to graft and patient survival rates. This means that trials examining these directly clinically relevant endpoints would require unfeasibly large numbers of patients, and don't fit within conventional grant funding envelopes.

Transplantation trials, therefore, mostly rely upon surrogate endpoints, which by definition have limitations. These surrogates must be robustly associated with real-world clinically-relevant outcomes, which necessitates the use of carefully-designed registries. Surrogate endpoints must be sensitive, and well-distributed continuous variables. Conventional definitions of early allograft function endpoints across organs can often be improved upon—for example binary classification of kidney transplants as resulting in delayed or immediate graft function hides much detail, as do various definitions of early allograft dysfunction in liver transplantation. Examples of candidate endpoints that should be the focus of further validation work include: (i) AI-assessment of MRCPs to produce a continuous index, rather than a clinical binary classification of non-anastomotic strictures versus none (72); (ii) the use of MEAF rather than EAD for early liver function (73); functional assessment by glucose tolerance testing in pancreas transplantation (74); and time to onset of graft function (e.g., tCr50) rather than the binary metric of delayed graft function (DGF) in kidney transplantation (75).

Given the protocol complexity and variability present in current perfusion research, and the importance of long-term registry data in validating novel research endpoints, a key focus for the next two decades will be establishing effective and efficient means for recording the details of transplant interventions and patient outcomes to a resolution sufficient for endpoint validation, and protocol reproduction. It is of paramount importance that interventions performed both as research and as a clinical service are clearly specified and fully reproducible. This is not straightforward in isolated organ perfusion research. Depending on the system being used, medications are often added ad-hoc, perfusate constituents changed, and perfusion parameters modified at the will of individual investigators. As these devices move beyond the role of simple machine perfusion and start to be used as delivery mechanisms for drug, gene or cell therapeutic interventions, medical regulators worldwide may begin to regard these systems within different regulatory categories. It is possible that new regulatory frameworks governing medical devices and drug-device combinations will need to emerge to deal with the use of complex combination perfusion devices. A central repository for organ perfusion protocols, which can be referenced both by individual publications and registries, would be prudent, as would the production of CONSORT-type reporting guidelines for perfusion studies. Direct data feeds from electronic patient record systems, and even from perfusion devices, through to registries and study data capture tools will need to be considered. Over time, as perfusion becomes more ubiquitous, the generation of a core outcomes set (COS) for each organ and type of perfusion would be of great use for standardisation, as has been seen in renal transplantation (76). Ensuring that isolated organ perfusion studies and clinical implementations are adequately regulated and recorded will be critical to the success of the field over the coming years.

Finally, the field will need to accommodate new trial methodologies in order to accommodate and optimise this heterogeneity and protocol complexity. Conventional two-arm randomised controlled trials are impractical for optimising individual perfusion parameters or drug additions along hundreds of potential dimensions. With adequate reporting and standardisation, registry analyses and historically controlled single-arm trials may become increasingly useful. Threshold-crossing designs, where single-arm trials are used to triage new interventions to adoption (if results are unequivocal), further randomised investigation (if promising but equivocal), or termination (if futile) are a formalisation of these ideas which should be promoted. Platform trials which encompass several interventions and leverage common control groups are another mechanism for increasing research efficacy and rate of progress. Prospective trials of ex-situ organ assessment require special consideration, and stepped-wedge designs where new technologies are made available sequentially to different centres may be particularly useful. The future of isolated organ perfusion over coming years will be shaped by the adoption of these ideas and designs.

## Trial objectives & intervention

The objectives of isolated organ perfusion over the next 15 years will evolve alongside advancements in bioengineering and personalised medicine. The primary goal will continue to be improvement of transplantation outcomes, however as organ perfusion technology advances we are likely to see its role extend into drug development and discovery, investigation of organ-specific pathophysiology, and patient-connected applications such as extracorporeal organ support and *in situ* isolated organ perfusion for delivery of therapeutics.

Many of the transplantation-related goals of isolated organ perfusion are organ specific, depending on the specific challenges in transplantation of that organ. However, there are common themes across all organs. These include the minimisation of ischaemia during preservation, viability testing prior to transplant, therapeutic interventions to improve graft quality, and strategies to achieve immunological tolerance (or hypo-responsiveness) in the recipient.

With increasing donor comorbidities (age, cardiovascular disease, diabetes, obesity), the average quality of available organs for transplantation is diminishing. Over the next 15 years innovations in perfusion technology and our understanding of isolated perfused organ physiology may allow treatment and repair of damaged organs, regeneration of chronically impaired grafts and accurate assessment for clearer prediction of post-transplantation graft function. The optimum matching of patient and donor will benefit from the use of artificial intelligence to combine the input of all relevant factors, including conventional donor and recipient parameters as well as graft performance during machine perfusion, set in the context of data and outcomes of past transplants. This will enable much more accurate risk assessment to determine which patient (if any) should be offered a particular organ, and will provide better information to help patients. This will significantly increase organ utilisation by increasing the use of higher-risk organs, and direct organs to the patients most likely to benefit. In doing so this will reduce waiting list mortality and increase the provision of organ transplantation to a wider pool of patients.

In liver perfusion, the primary goal will be improving the prediction of and treatments to prevent post-transplantation biliary complications which remains a major limitation in the use of higher-risk livers. Machine perfusion offers a platform to minimise injury to the biliary epithelium and potentially stimulate its regeneration prior to implantation. However, the precise pathophysiology that leads to long-term biliary complications is not fully understood. As a result, translating short-term interventions during perfusion into meaningful improvements in long-term biliary outcomes remains a challenge. Interventions undergoing preclinical assessment include senescence-directed therapies, cellular therapies to improve biliary regeneration and refined perfusion technologies to manage the transition of temperature and oxygenation that is thought to contribute to IRI.

For the kidney, accurately predicting long term graft function and extending the longevity of graft function will be key aims of machine perfusion. Chronic rejection, disease recurrence and progressive graft fibrosis are today's main challenges in terms of long-term kidney transplant outcomes. Perfusion may allow for routine assessment of organ quality prior to transplantation by monitoring biomarkers. In addition, early interventions with protective agents that reduce inflammation, promote endothelial repair or modulate fibrotic signalling pathways may prolong organ lifespan significantly (77).

In pancreas transplantation, graft loss due to pancreatitis secondary to IRI remains one of the most significant challenges. Isolated pancreas perfusion may allow for real-time assessment of perfusion dynamics and endothelial integrity, enabling early identification of organs at high risk for IRI. In addition, perfusion platforms may support delivery of protective agents to reduce the risk of graft loss. Finally, pancreas perfusion may be used to improve the quality and quantity of islet cell isolation. Pancreas transplantation is the area with the least evidence for the application of perfusion: it has proved to be a more challenging environment that other organ types, particularly in the context of NMP, where there is no published report of successful human transplantation after perfusion.

In heart transplantation, minimising ischaemia-reperfusion injury and the detrimental effects of cold storage will be a key priority. Machine perfusion enables continuous myocardial perfusion during preservation, but real-time functional assessment is currently limited. Future iterations of cardiac perfusion devices may provide robust volumetric assessment of contractility and stroke volumes. In time, machine perfusion may allow metabolic reconditioning and potential pharmacologic interventions to minimise primary graft dysfunction. Machine perfusion also expands preservation windows, which as the PROCEED II trial demonstrated is particularly important in heart transplantation given the vulnerability to ischaemia causing significant logistic constraints.

In the lung, pro-inflammatory cascades leading to primary graft dysfunction are a leading cause of early morbidity and mortality and contribute to the development of chronic graft dysfunction and organ failure. Isolated lung perfusion allows functional assessment of marginal lungs in terms of gas exchange, compliance and vascular resistance. Perfusion platforms have already been used to treat lungs with poor gas exchange and even infections, in order to improve these measured parameters and enable successful transplantation.

## Implementation of organ perfusion

The anticipated advances in organ perfusion technology and science will increase the number of organs perfused and the duration over which they are perfused. As discussed, the potential for detailed organ assessment and effective intervention between donation and implantation is predicted to increase. To translate these developments from clinical research to routine clinical use, centralisation of organ perfusion is very likely to be required. Such “assessment and reconditioning centres (ARCs)” would provide the necessary infrastructure, expertise and resources to support 24/7 organ perfusion, as well as advanced organ assessment and intervention.

**Figure 1:**
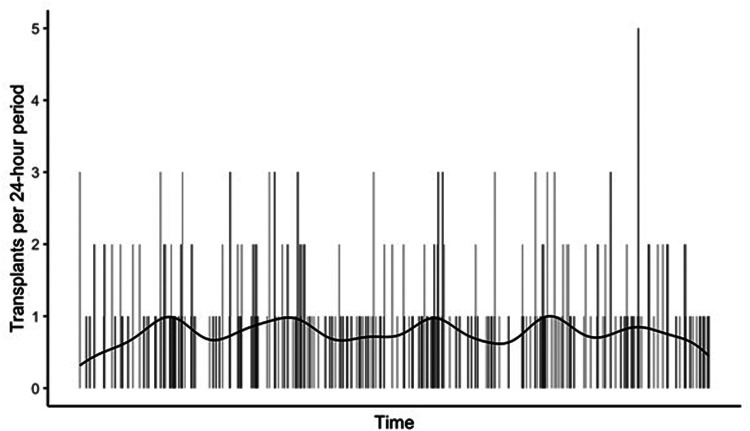
Concept as an ARC as capacitor, using data from Oxford transplant centre (2020–2022). The vertical bars describe the current random frequency of organ transplants. By acting as a “capacitor”, the combination of an ARC system and reliable prolonged perfusion could shift the pattern to the solid line, improving logistics and increasing the total number of transplants that are possible given a fixed maximum operating capacity. Demonstrating that a unit like Oxford doing more than 200 transplant per year would produce an average of 0.3–1 transplant per day. Meaning that without increasing unit infrastructure one could more reliably increase the annual transplant output to closer to 350, with at least 1 transplant per day.

The perfusion strategy for a donor organ before, during and after the ARC will be individualised to the graft. Grafts may undergo prolonged perfusion (i.e., 24 h or longer) to support logistical considerations. Within limits, prolonged perfusion will enable the ARC to act as a “capacitor” by delivering organs to implanting centres when needed—when organ supply is greater than demand, organs are “stored” at the ARC for longer; when transplant centres have the capacity to transplant organs are then “released”. This will optimise implanting centre resource utilisation, Figure 1 demonstrates hypothetical modelling of the concept with data from the Oxford Transplant Centre. The use of operating department infrastructure could be planned to increase the proportion of implants occurring during the day, to reduce overnight staffing costs and improve workforce sustainability. The capacitor function of the ARC could even improve graft supply for emergency indications (e.g., liver transplantation for primary non-function) and allow more time for recipient transport and preparation for surgery.

Detailed organ assessment during perfusion will extend the assays required beyond the currently used perfusion haemodynamics and point-of-care tests of metabolic function. These assays could include specific perfusate/tissue biomarkers, histological/immunohistochemical analysis, and radiological assessment. To facilitate timely organ assessment, the capacity to perform these assessments should be available either at the ARC (e.g., enzyme-linked immunosorbent assays (ELISAs) for specific biomarkers; tissue sample processing, staining and scanning) or in close conjunction with local hospitals (e.g., interpretation of histological images; MRI scanning). The potential for organ reconditioning during organ perfusion could include small-molecule drug delivery, such as anti-microbials, defatting of steatosis grafts, immunomodulation or gene therapy. As with organ assessment, “off-the shelf” treatments like small molecules or gene therapies such as siRNA, should be available at the ARC with the necessary local experience to deliver. More complex techniques such a recipient-derived cell therapies, such as in the TWO study (78), which are currently limited to living donor recipients could but utilised in the deceased donor setting, in collaboration with hospitals local to the ARC.

To enable this sophisticated approach to centralised organ perfusion, staffing of an ARC would need to include the following roles: (i) organ perfusionist (management of prolonged perfusion, sample collection, delivery of organ-directed treatment); (ii) laboratory technician (biomarker and histological analysis, preparation of interventions); (iii) surgeon, as an extension of their role at local hospitals (organ backbench preparation, cannulation, biopsies); (iv) co-ordinator (arranging transport services and liaising with donor/recipient centres). Careful coordination and appropriate infrastructure will be particularly important—the introduction of complex organ assessment and intervention techniques would mean that organs could be accepted pre-perfusion, intra-perfusion or post-perfusion, necessitating a dedicated organ co-ordinator closely associated with the ARC.

The number and location of ARCs should reflect the local conditions, considering both population and geography. Ideally ARCs should be situated so that organ transport times can be limited both from donor hospitals and to transplant centres. For example, we might envisage that 3–4 ARCs would be required to support a retrieval service the size and population of the United Kingdom [4,570 transplants 2023–2024 (79)]. ARCs may be associated with local transplant centres to take advantage of existing infra-structures (surgeons, histology analysis, radiology), but independent and centrally run. In the US, it is likely that the ARC function will be based at Organ Procurement Organisations (OPOs).

## The future of health economics for organ perfusion

The potential impact of perfusion technology, and in particular assessment and intervention delivered in centralised ARC facilities, on broader transplant health economics is not well researched. During the development of machine perfusion technologies efficacy has been the primary concern, with economic and health technology assessment becoming a focus more recently. More efforts are needed to evaluate the health economics associated with older and newer technologies, and to question how these interventions interact with conventional solid transplantation indications, organ matching, and transplantability thresholds. Technological advances in organ preservation have the potential to transform existing geographic inequity and enable wider organ assessment and sharing. It could be that machine perfusion could reduce overall transplantation costs worldwide, leveraging longer preservation times without organ deterioration, better organ assessment, and improved organ/recipient matching translating to increased utilisation and improved outcomes (80, 81), but this will be highly dependent on the organ and perfusion type, with current economic models failing to be unanimous (Table 2).

**Table 2:** Summary of key econmic analyses related to perfusion technologies, highlighting significant variation in cost, but with braodly speaking favourable outcomes for the cost effectiveness of the technolgies.

Organ/modality	First author & year	Country & study type	Perspective & horizon	Comparator	Sample size feeding the model/analysis	Funding source	Main economic result
Liver—HOPE	Endo, 2025 (82)	Netherlands—within-trial CU-analysis	Hospital payer; 12 months	Static cold storage (SCS)	70 transplanted recipients (35 HOPE, 35 SCS)	Public—(Dutch Transplant Foundation)	HOPE dominant: mean cost €35,900 vs. €43,300 (–€7,400) and slightly higher QALY; 79% probability of cost-saving
	Zimmermann, 2022 (83)	UK—de-novo Markov model)	NHS payer; lifetime	SCS; NMP	Literature pool **≈**870 transplant cases (9 HOPE/D-HOPE studies)	Public—(NIHR)	Base-case ICER £204,000/QALY—HOPE *not* cost-effective at £30k threshold unless utilisation ↑ and EAD ↓ by ≥50%
Liver—NMP	Javanbakht, 2020 (84)	UK—company Markov model (OrganOx Metra)	NHS; lifetime	SCS	220 recipients (COPE RCT)	Industry—(OrganOx Ltd)	ICER **£**8,300/QALY—99% chance cost-effective at £20 k
	Zimmermann 2022 (83) (same group)	UK—public model	NHS; lifetime	SCS; HOPE	Same systematic-review pool (HOPE 870; NMP 340)	Public—(NIHR)	NMP cost-effective in only 3% of PSA draws owing to high device cost
	NICE MIB 275 (2021)	UK—per-procedure budget impact briefing	Provider (NHS); per case	–	Expert panel—no patient data	Public—(NICE)	Consumables + lease ≈£7,700 per case; cost-neutral only if ≥25 extra livers transplanted annually
	Wehrle, 2024 (85)	USA—multicentre retrospective matched-cohort cost study (“back-to-base” NMP)	Hospital/provider; 90-day costs	Matched SCS controls	Risk-matched analysis on **1**55 NMP (118 DBD + 37 DCD) vs. 310 SCS	Academic -(Cleveland Clinic)	Despite higher device cost, no increase in 90-day total cost; lower complication-derived costs offset acquisition
Kidney—HMP (non-oxygenated)	Groen, 2012 (86)	Netherlands/Belgium—economic arm of paired-kidney RCT	Hospital & societal; 3 yr + lifetime Markov	SCS	336 kidney pairs (672 grafts)	Public—(EU FP6/Dutch MoH)	HMP dominant: better outcomes and –€3,300 mean lifetime cost
	Wight, 2003 (87)	UK—decision model + meta-analysis	NHS; lifetime	SCS	16 RCTs totalling **≈** 3 800 grafts	Public—(NIHR HTA)	HMP cost-saving in 80% of NHBD scenarios, 50%–60% in DBD
	Tedesco-Silva, 2024 (88)	Brazil—Markov budget-impact & cost-effectiveness model	Public payer; 5 yr	SCS	Model cohort built from national registry data: 27 613 patients on waiting list; annual recovery ∼1,586 kidneys (19% ECD)	Public—(Brazilian SUS)	Selective HMP for ECD kidneys would add 1,123 extra transplants, cut waiting list by 815 and waiting-list deaths by 120; 5 yr budget impact + US $4.45 M

Machine perfusion use in organ preservation for transplantation is evolving with some applications still in early-phase clinical trials, and very little clinical evidence beyond the stage of simple perfusion, rather than perfusion-based interventions. Rigorous health economic analyses based on robust estimates of effect size will be needed to assess the true value of these novel devices and applications, and which of these move forward to wide-scale implementation and guideline recommendation. New healthcare technologies face economic challenges even in high-income countries, and the need to prove benefit at an affordable cost is increasingly part of the development of any new healthcare modality.

Cost-effectiveness calculations vary across healthcare systems, and also depending on the organ and type of perfusion (e.g., renal HMP is far cheaper than cardiac or liver NMP). Cost effectiveness is complex to assess and specific to individual healthcare systems, especially in an environment as complex as organ transplantation. Even within Europe, differences between countries are important: Endo et al. conducted a cost analysis of the Dutch DHOPE-DCD RCT and showed HOPE to be cost-effective in DCD transplant, reducing total medical costs up to 1 year post-transplant (82), but a study from the UK economic modelling found HOPE to be much more costly, £204,059 per QALY, outside the willingness to pay threshold of £30,000 (83). Using the same UK model liver NMP was estimated to cost over £1,000,000 per QALY, whereas another analysis costed the same NMP in a UK setting at £8,300 per QALY (84). This large variation in modelling makes it difficult for national healthcare payers to decide how to implement these technologies (Table 2).

The current pricing of devices and their disposable sets is high, for both HOPE and NMP. In the US, disposable sets cost in excess of $30,000, which can raise issues with reimbursement depending upon institution and insurer. Differential pricing is normal between different regions; this reflects wide variations in the service model used in different territories as well as large variation in the cost of delivering a service. Clearly the cost of machine perfusion (including the cost of delivering it) is high, and this is a limiting factor in lower income countries and less well-funded healthcare environments. As with most technologies, over time and with increasing volume cost will come down. On the journey to 2040 we anticipate increasing uptake of the technology, refinement in the accuracy of economic cost models, and increasing integration of perfusion into national healthcare systems, as has already happened with renal HMP/HOPE in the US, France, Belgium and the Netherlands.

Equity in accessing transplantation services is far from a reality at present and this is a recognised failing in the existing system. Expensive technologically advanced solutions to the challenges of transplantation will exacerbate this inequity if our health economic modelling does not account for this need for equity. Single-payer systems like the UK's NHS typically evaluate health technologies through centralised health technology assessment bodies, focusing on population-level cost-effectiveness. In contrast, the US system has variable adoption driven by a mix of transplant centre competition, private insurance, and Medicare policies. By 2040 the health economics of ex-situ organ perfusion are likely to look very different, perhaps including frameworks for dynamic value-based pricing where real-world performance data will adjust reimbursement.

## Conclusion

By 2040, isolated organ perfusion will have evolved from a preservation adjunct into a fully integrated platform for organ repair, assessment, and tailored therapy. The cumulative effect of advances in hypothermic and normothermic technologies, AI-driven biomarkers, and centralised ARCs will improve graft utilisation, and enable delivery of complex interventions. Robust registries and adaptive trial designs will should facilitate high-quality evidence, while dynamic, value-based reimbursement models might determine their sustainable adoption across diverse health-care systems. Realising this future will require continued collaboration between engineers, clinicians, regulators, and economists, and will ultimately result in a transplantation paradigm defined less by organ scarcity and uncertainty, and more by equity and personalised graft management.
